# *Rhipicephalus turanicus*: from low numbers to complete establishment in Cyprus. Its possible role as a bridge-vector

**DOI:** 10.1186/1756-3305-7-S1-P11

**Published:** 2014-04-01

**Authors:** D Chochlakis, I Ioannou, B Papadopoulos, Y Tselentis, A Psaroulaki

**Affiliations:** 1 Regional Laboratory of Public Health of Crete, Heraklion, Crete, Greece; 2 Veterinary Services, Nicosia, Cyprus; 3 Laboratory of Clinical Bacteriology, Parasitology, Zoonoses and Geographical Medicine, University of Crete, Heraklion, Crete, Greece

## 

We describe herein the abundance of *Rhipicephalus turanicus *in Cyprus, and its potential role as bridge vector carrying tick-borne pathogens among various hosts.

Following the first recording (1970-72), at low numbers, of *R. turanicus *in Cyprus, two studies took place (March 1999 - March 2001, January 2004 - December 2006), during which, ticks were collected from ruminants (goats, sheep, bovine), dogs and wild mammals (foxes, wild-rabbits and mouflons) from different sites of the island. All ticks were morphologically identified to the species level, washed in 70% alcohol, rinsed in sterile water, dried on sterile filter paper and triturated individually. Following DNA extraction [QIAampTissue Kit (QIAGEN, Germany)] all ticks were tested for *Rickettsia *species and *Coxiella burnetii *by Real-time PCR targeting the *gltA *gene and IS1111 insertion sequence, respectively, and for *Anaplasma *species by PCR targeting the 16s rRNA gene. Samples positive for *Rickettsia *species were further amplified by PCR targeting the *ompA *and *ompB *genes.

Of the 3950 ticks collected, 805 (20.4%) were identified as *R. turanicus*; this species was the only one, amongst the rest of the ticks (*R. bursa*, *R. sanguineus*, *Hyalomma anatolicum excavatum*, *H. marginatum*, *H. m. rufipes*, *Ixodes gibbosus*, *I. ventalloi*, *Haemaphysalis sulcata*, *H. punctata*), that was collected from every host. Of the ticks tested, 125/805 (IR: 15.5%) were tested positive for *Rickettsia *species [*R. massilliae *(83/125), "Candidatus *Rickettsia barbariae*" (5/125), "Candidatus *Rickettsia barbariae *genotype Cretocypriensis" (26/125) and "*Candidatus Rickettsia tselentii*" (11/125)]. The latter *Rickettsia *species showed a close relationship with *R. africae*, was described for the first time in Cyprus and was identified in *R. turanicus *only disregarding the animal host. Of the 805 ticks, 107 (IR: 13.3%) were tested positive for *C. burnetii*, and 16 (IR: 2%) were positive for *Anaplasma *species. Five ticks harboured both *Rickettsia *species and *C. burnetii*.

*Rhipicephalus turanicus*, largely distributed in the Mediterranean sub-region, Africa and Asia, is flexible and adaptable and can be found in a rich variety of hosts (domestic and wild). It has well adapted and spread over a great variety of animal species in Cyprus during the last 40 years and seems to play an importance role as a bridge-vector of tick-borne pathogens. The changing climate, geographical position of Cyprus (wintering area for migratory birds), make the island an important cross-over for possible dispersal of ticks and their tick-borne pathogens from East to the West.

